# Patients’ and care partners’ views on communicating the cause of dementia and related uncertainties: A qualitative study

**DOI:** 10.1177/13872877261435882

**Published:** 2026-04-03

**Authors:** Jolanda H. M. Dobbe, Leonie N. C. Visser, Tim I. Waagemans, Geert J. Biessels, Flora H. Duits, Marij A. Hillen, A. W. Evelien Lemstra, Gerwin Roks, Inez H. G. B. Ramakers, Ellen M. A. Smets

**Affiliations:** 1 Department of Medical Psychology, 26066 Amsterdam UMC location AMC, Amsterdam, the Netherlands; 2 Alzheimer Center Amsterdam, Neurology, Vrije Universiteit Amsterdam, Amsterdam UMC location VUmc, Amsterdam, the Netherlands; 3 Amsterdam Public Health, Quality of Care, Amsterdam, the Netherlands; 4 Amsterdam Public Health, Ageing and later life, Amsterdam, the Netherlands; 5 Division of Clinical Geriatrics, Center for Alzheimer Research, Department of Neurobiology, Care Sciences and Society, 27106 Karolinska Institutet, Stockholm, Sweden; 6 Amsterdam Public Health, Personalized Medicine, Amsterdam, the Netherlands; 7 Department of Bioethics and Health Humanities, Julius Center for Health Sciences and Primary Care, 8124 University Medical Center Utrecht, Utrecht University, Utrecht, the Netherlands; 8 Department of Neurology, UMC Utrecht Brain Center, 8124 University Medical Center Utrecht, Utrecht, the Netherlands; 9 Amsterdam Neuroscience, Neurodegeneration, Amsterdam, the Netherlands; 10 Department of Neurology, ETZ Hospital, Tilburg, the Netherlands; 11 Alzheimer Center Limburg, School for Mental Health and Neuroscience (MHeNs), Maastricht University, Maastricht, the Netherlands

**Keywords:** Alzheimer's disease, dementia, dementia with Lewy bodies, diagnosis, frontotemporal dementia, mild cognitive impairment, vascular dementia

## Abstract

**Background:**

Dementia is a syndrome with various presumed causes, such as Alzheimer's disease. Communicating the cause of dementia is challenging for clinicians, involving complex information and frequent uncertainties.

**Objective:**

We explored perspectives of patients with mild cognitive impairment or dementia (Alzheimer's disease, Lewy body dementia, frontotemporal dementia, vascular dementia, or mixed pathology) and their care partners, on what communication they prefer about the cause of dementia and related uncertainty.

**Methods:**

We held focus groups and interviews in the Netherlands, with 22 patients who had received a diagnosis at the memory clinic in the past four months, and their 21 care partners. Data were analyzed using content analysis.

**Results:**

Participants showed poor knowledge of the presumed cause, often mentioning risk factors (e.g., age, “bad luck”, genetics, lifestyle). During the diagnostic disclosure, clinicians’ communication efforts (e.g., visualization of test results) enhanced participants’ understanding. Participants’ needs regarding etiological information varied: some desired detailed information to obtain clarity and receive appropriate care, while others preferred minimal information because of perceived limited added value and potential information overload. We observed no clear demographic or clinical patterns in these preferences. Participants emphasized the importance of clinicians being explicit about the presence of uncertainty, with care partners particularly indicating strong information needs.

**Conclusions:**

Findings reveal much variation in the extent to which patients and care partners wish to receive information about the cause of dementia and related uncertainty, highlighting the need for a personalized approach.

## Introduction

Dementia is a clinical syndrome with many potential causes, with Alzheimer's disease (AD) being the most common, followed by vascular dementia (VaD), Lewy body dementia (LBD), and frontotemporal dementia (FTD).^1,2^ From a clinician's perspective, determining the cause of dementia allows better prediction of disease progression, and could help to identify and apply disease-modifying treatment and other interventions to delay the disease, which is therefore also of importance for patients.^3–5^ The perceived importance of the cause of dementia has driven substantial efforts and progress in the development of diagnostic tests to determine such a cause.^3,5^

Nevertheless, identifying the underlying cause is challenging. Determining non-AD causes is difficult, as clinical symptoms often overlap with AD, and specific biomarkers for non-AD causes are less developed.^6–9^ Another important challenge is the occurrence of mixed pathology, indicating the coexistence of multiple etiologies that cause someone's symptoms.^5,10–13^ Current diagnostic guidelines often focus on one etiology, whereas in practice a combination of etiologies may be present and various test results need to be combined and interpreted together.^14^ Lastly, test results can be ambiguous, i.e., being borderline abnormal or conflicting, thereby complicating their interpretation.^15^ Thus, determining the cause(s) in persons with mild cognitive impairment (MCI) or dementia is complex, involving many uncertainties.

Consequently, clinician-patient communication about diagnostic test results and etiological information becomes increasingly difficult. As the diagnostic test battery expands, clinicians need to share increasing amounts of information with patients during diagnostic consultations, while their available time for each patient is limited.^16,17^ Although clinicians often establish the cause of dementia (e.g., dementia due to AD), they may choose to communicate only a broad clinical syndrome (dementia), assuming that detailed etiological information could cause confusion or distress.^18,19^ However, patients have indicated a need to know and understand their diagnosis, including the etiology.^18,20^ An additional difficulty for clinicians may be communicating uncertainty when explaining diagnostic test results. A previous analysis of audio-taped post-diagnostic testing consultations found that in a vast majority, clinicians expressed uncertainty related to patients’ diagnosis or the etiology thereof, disease progression and conflicting test results.^15^ The uncertainties that were discussed were variable in nature, ranging from probability (i.e., the inability to predict the future), to ambiguity (i.e., results being incomplete, indefinite, tentative or inconsistent) and complexity (i.e., difficulty in aspects of the phenomenon itself, like multicausality or unpredictability of disease development).^21^ Explaining these uncertainties is challenging for clinicians, and difficult for patients and care partners to comprehend, which might influence their wellbeing and coping with the disease.^22^ To date, recommendations on how clinicians can optimally communicate about uncertainty in the context of etiological test results for dementia are lacking. Some strategies to communicate uncertainty were proposed as helpful in disclosing prognostic information in the field of oncology, such as using a three-step approach (normalizing uncertainty, addressing emotions and supporting with coping) or providing precise information about the uncertainty, like numerical estimates.^23,24^ Yet it is unknown if and to what extend individuals who visit the memory clinic understand etiological information and value these communication strategies.

This qualitative study aims to investigate the views of patients with MCI and dementia, and their care partners, on what they prefer to be communicated about the cause of dementia and related uncertainty, and how this could be communicated.

## Methods

### Study design

This qualitative study comprises interviews and focus group sessions with patients with MCI and dementia, and their care partners, i.e., the person(s) accompanying the patient to the diagnostic consultation(s). This methodology was used to obtain in-depth insight into their views.^25^ The study was part of a larger research project (Timely, Accurate, and Personalized (TAP)-dementia), that strives to improve the diagnosis of all forms of dementia and the communication thereof.^26^ The study was exempted from formal approval by the ethics committee of the Amsterdam University Medical Center, according to the Dutch law (2023.0382). The Consolidated criteria for reporting qualitative studies (COREQ) were followed.^27^

### Context

In the Netherlands, memory clinics are present in approximately 70% of hospitals.^28^ Approximately half of patients visiting a memory clinic receive a diagnosis of dementia, while about one quarter are diagnosed with MCI or subjective cognitive decline. Most memory clinics follow the Dutch multidisciplinary guideline Diagnostics and Treatment of Dementia, with the diagnostic work-up typically including neuropsychological assessment, neuroimaging and blood testing.^29^ Some memory clinics have a more extensive diagnostic work-up, also including tests like EEG and lumbar puncture.^30^ In addition, patients increasingly have access to their electronic medical records, often allowing them an opportunity to review diagnostic test results prior to consultation.

### Participant recruitment and procedures

Patients with MCI^31^ and dementia (due to AD, LBD, FTD, VaD, or mixed pathology),^32–36^ and their care partners were recruited from three memory clinics in the Netherlands, of which two were academic (i.e., university-affiliated hospitals). One of these academic centers had an extensive diagnostic work-up as previously described. Inclusion criteria were that the patient: 1) had undergone diagnostic testing and received a diagnosis in the past four months; 2) was able to verbally communicate and provide informed consent (based on the clinician's judgement); and 3) understood that a broad clinical syndrome diagnosis had been made (based on medical history reported in medical file). Patients and care partners with poor comprehension of the Dutch language and care partners younger than 18 years old were excluded. Clinicians first informed eligible patients and their care partners about the study during the consultation in which a diagnosis/diagnostic information was shared (‘diagnostic consultation’). If verbal consent was obtained, the researcher contacted either the patient or care partner by phone, to provide further study information. We purposively sought to include variation in causes of dementia to investigate whether perspectives differed.

Focus group sessions and interviews were held between December 2023 and January 2025. Patients and care partners could participate together or alone. Focus group sessions were conducted at the memory clinic where patients had had their diagnostic consultation. We decided to include a maximum of three patients with the same dementia cause and three care partners per session.^37–39^ Alternatively, patients and care partners could choose to be interviewed if they felt that participating in a focus group was too overwhelming, or when inclusion in a focus group would result in a too long time-interval between diagnosis and the session. Since the latter often occurred, a pragmatic decision was made to no longer offer focus groups. The interview took place at the memory clinic or at participants’ home, through phone or Microsoft Teams, based on participants’ preference. Each focus group session and interview was led by a facilitator (JD (female health scientist) or TW (male clinical neuropsychologist); both not involved in the diagnostic process) and an observer (JD or TW) who took notes. Sessions were audio recorded and had a maximal duration of 90 min. Prior to the sessions, participants signed informed consent and completed a questionnaire assessing sociodemographic and medical characteristics. Lastly, broad clinical syndrome diagnosis and dementia cause, and scores on the Mini-Mental State Examination (MMSE)^40^ and Montreal Cognitive Assessment (MoCA),^41^ were retrieved from patients’ medical records.

### Interview- and focus group guide

A semi-structured topic guide (see Supplemental File 1) was used for both focus group sessions and interviews. The topic guide comprised two main components; 1) discussing patients’ and care partners’ understanding of the dementia cause and what had been communicated about this during the diagnostic consultation; and 2) hypothetical scenarios of how clinicians may communicate about different types of uncertainty related to etiological information. For the first component, participants were invited to reflect on five statements that depicted different aspects of communication about the cause (i.e., amount and understandability of information, feelings of (un)certainty, emotions and degree of detail), based on previous research.^42,43^ For the second component, three visualizations of hypothetical scenarios were presented of a conversation between a clinician and patient, varying in presence and type of uncertainty regarding the dementia cause (see Supplemental File 2).^15,44^ In scenario 1, the cause was certain. Scenario 2 included uncertainty about the cause due to complexity, by presenting an example of suspected mixed pathology. Scenario 3 included uncertainty due to ambiguity, as test results did not fit a specific dementia cause. For each scenario we asked participants what their reaction would be if receiving this information, and what questions the scenario raised. Subsequently, for scenarios 2 and 3 we provided two communication approaches of the clinician. The first approach consisted of a three-step approach for communicating uncertainty (i.e., normalizing uncertainty, addressing patients’ emotions, and supporting with coping).^23^ The second approach included a (more) detailed explanation of the clinician of what the uncertainty entailed.^24^ Participants were asked whether these approaches would enhance their understanding of the diagnostic information. The scenarios were adjusted according to the patient's dementia cause.

### Analyses

Audio recordings of the interviews and focus group sessions were transcribed verbatim by TW. Thereafter, all transcripts were independently coded by two researchers (JD, TW) using conventional content analysis, meaning that categories and themes were identified based on the data.^45^ MAXQDA 2022 software was used for both transcription and coding of the data.^46^ JD and TW started by reading a transcript, and by creating codes that reflected their key thoughts. After coding a transcript, codes were discussed during consensus meetings. This process was repeated for a number of transcripts, which resulted in a coding scheme. Subsequently, the remaining transcripts were coded using this coding scheme and new codes were added when necessary. Another member of the research team (ES) was consulted halfway, to enhance researcher triangulation. When all transcripts were coded, JD and TW independently generated themes based on the coding scheme and visualized these in a thematic map. Consequently, their thematic maps were discussed and combined into one map, which was discussed within the research team (JD, TW, ES, LV), to decide on definite themes. Illustrative quotes were selected from the transcripts by JD and TW, removing any information that could refer to a specific clinician/patient. We used descriptive statistics to report characteristics of the participants.

## Results

### Participant characteristics

We performed one focus group (three patients with MCI/dementia due to AD and two care partners) and 21 interviews with 19 patients and 19 care partners (16 interviews patient together with care partner(s); three interviews patient only; two interviews care partner only). Characteristics of the participants are reported in Table 1. Most patients had dementia as broad clinical syndrome diagnosis (N = 17, 77%). As underlying cause, seven (32%) patients had AD. Four patients had mixed pathology, including two with AD and LBD, one with AD and FTD, and one with AD and VaD. Half of the patients were referred directly to the memory clinic by their general practitioner, while the other half visited the hospital for a second opinion.

**Table 1.** Characteristics of the participants.

Characteristics	Patients (N = 22)	Care partners (N = 21)
Age, mean ± SD	69 ± 8	67 ± 10
Female, n (%)	11 (50)	13 (62)
Relation to the patient, n (%)		
Husband/Wife/Life partner	n/a	19 (90)
Child	n/a	2 (10)
Education, median (IQR) ^a^	4 (2–7)	5 (4–7)
Living situation, n (%)		
Living at home, alone	3 (14)	1 (5)
Living at home, together	19 (86)	20 (95)
Broad clinical syndrome diagnosis, n (%) ^b^		
MCI	5 (23)	6 (29)
Dementia	17 (77)	15 (71)
Cause of dementia, n (%) ^b^		
AD	7 (32)	7 (33)
LBD	3 (14)	4 (19)
FTD	6 (27)	6 (29)
VaD	2 (9)	2 (10)
Mixed ^c^	4 (18)	2 (10)
MMSE, mean ± SD ^d^	25 ± 3	n/a
MoCA, mean ± SD ^d^	22 ± 3	n/a
Time since diagnosis, n (%)		
0–1 month	3 (14)	2 (10)
1–2 months	7 (32)	4 (19)
2–3 months	6 (27)	8 (38)
3–4 months	6 (27)	7 (33)
Hospital, n (%)		
Academic	20 (91)	17 (81)
Non-academic	2 (9)	4 (19)
Second referral, yes, n (%)	11 (50)	11 (52)

^a^ Education is rated using the Dutch Verhage system, ranging from 1–7.^47^

^b^ This concerns data of the patients.

^c^ Mixed pathology included: 1× AD + FTD, 2× AD + LBD and 1× AD + VaD.

^d^ 20 patients had a MMSE score, 19 patients had a MoCA and 17 patients both had a MMSE and MoCA.

AD: Alzheimer's disease; FTD: frontotemporal dementia; IQR: interquartile range; LBD: Lewy body dementia; MCI: mild cognitive impairment; MMSE: Mini-Mental State Examination; MoCA: Montreal Cognitive Assessment; SD: standard deviation; VaD: vascular dementia.

A total of seven themes, divided over two categories, emerged from the focus group and interviews. These themes are further described below.

### Patients’ and care partners’ understanding and communication preferences regarding the cause of dementia

1. *Limited understanding of the meaning of a dementia cause*

When participants were asked about possible causes of their symptoms, they rather suggested risk factors for dementia than a dementia cause (e.g., dementia due to AD), implying that the meaning of ‘dementia cause’ was unknown to them. Factors like age, “bad luck”, genetics and medication use were frequently mentioned. Additionally, some patients mentioned that always having lived healthy had not prevented the disease from occurring. Participants rarely distinguished between broad clinical syndrome diagnosis and the cause of dementia. They often used dementia and AD interchangeably, and were not aware of the difference. We observed the following interaction between a patient with dementia due to AD and her care partner:

*Patient: “So there is Alzheimer's and then dementia?”*

*Care partner: “No. No. Alzheimer's and dementia is the same. But the cause is Alzheimer's disease.”*

*Patient: “But what's the difference then? I don’t understand.”*

Notably, participants’ understanding sometimes changed during the conversation, mainly when they were shown scenarios of uncertainty in which broad clinical syndrome diagnosis and dementia cause were differentiated. Moreover, some participants indicated that clinicians could not determine the cause of the symptoms, that their clinician did not provide such information, or they did not remember whether the information was provided.

1. *Factors affecting participants’ expectations of the potential cause*

Participants’ expectations of the cause prior to entering the diagnostic consultation seemed dependent on several factors. First, participants frequently referred to the stereotypical dementia picture, namely dementia due to AD with substantial memory problems, which led them to consider non-AD causes less often. Second, various sources of information were generally consulted prior to the diagnostic consultation, such as the patient's medical record and online information. A majority of the participants mentioned disadvantages of having insight in test results and/or diagnosis prior to the diagnostic consultation, such as having difficulty understanding the information due to jargon, and information causing anxiety, worry, overthinking and confusion. The following quote also illustrates possible downsides of timely insight in the medical record:

*Care partner: “Well, I actually think that's very tactless of the hospital to put it [in the medical record] on the day before the consultation.”*

*Interviewer: “And, was that plain language?”*

*Care partner: “No, the language was very unclear, but with a little googling and I also have some medical knowledge myself…* *Then I discovered that things were not quite right after all. That was clear to me.” (Dementia due to AD)*

Conversely, some participants highlighted advantages of reading information in the medical record, such as the ability to prepare questions and being less overwhelmed when the diagnosis was disclosed. Of note, this mainly concerned participants who already (somewhat) expected the diagnosis. Furthermore, participants (mainly care partners) searched the internet for information about the symptoms and test results they had read in the medical record before the diagnostic consultation, which often resulted in additional concerns and obtaining irrelevant information. Finally, half of the participants visited the hospital for a second opinion, and therefore some of them already had presumptions of potential causes.

1. *Participants notice clinicians’ strategies to enhance understanding of etiological information*

During the diagnostic consultation, in the view of our participants, clinicians contributed to patients’ and care partners’ understanding of etiological information in various ways. For example, some participants mentioned that clinicians had described the relevance of performing a diagnostic test to confirm or eliminate a specific cause. Additionally, some clinicians had described how particular test results pointed to a certain cause, such as abnormalities of amyloid indicating AD. Moreover, participants mentioned visualization of test results, which they valued, for example when the clinician showed the MRI-scan or outlined and explained the most important results and the diagnosis on paper. In contrast, one care partner (who was a doctor himself) expressed dissatisfaction with communicating test results on paper because it did not match his and the patient's information need:

“*The clinician just gave a lecture for first-year students about Lewy Body, and drew it completely, I still have the drawings. I've been in school for a few years, but we didn't come for a lecture.” (dementia due to mixed pathology (LBD & AD), care partner)*

According to most participants, clinicians disclosed the dementia cause in a direct way (i.e., starting the consultation with sharing the dementia cause, then explaining the test results), which they generally appreciated since it was straight to the point and provided clarity. However, one frequently mentioned downside was that because of the prompt delivery and the need to process the bad news, subsequent information provided by the clinician was not always absorbed.

1. *Variable needs for information about the cause of dementia*

We observed variation in whether participants desired to receive information about the cause of dementia. On the one hand, some participants reported wanting to receive as much information as possible because information provided them more clarity, confirmed their suspicions, and allowed for receiving appropriate care. On the other hand, other participants provided reasons for not wanting to receive information about the cause. For example, some indicated that knowing the dementia cause adds little because disease progression cannot be prevented anyway, thereby highlighting the therapeutic irrelevance of the cause. Nevertheless, there was some variation in whether participants thought that the course of the disease differed based on the dementia cause as illustrated by the following quote:

*Care partner: “[patient] has never actually been that concerned with the form of dementia. We differ greatly in that [….]. [Patient] just says I have it and that's how it is. I don't want to say he isn't interested, because every type [of dementia] of course has its consequences. But he doesn't delve into that at all.”*

*Patient: “It would just make me sad, I say.” (mixed pathology (AD & LBD))*

Other reasons for not wanting etiological information were the risk of information overload during the diagnostic consultation, or the desire to search for information themselves. Lastly, a few patients indicated to have unfulfilled information needs after the diagnostic consultation since they mainly had questions related to prognosis and disease management rather than the diagnosis.

No clear differences were observed in information needs based on background characteristics (patients/care partners, age, education, dementia cause, hospital, second opinion). However, patients with mixed pathology and their care partners appeared particularly likely to want information about etiology. Moreover, patients and care partners from non-academic hospitals seemed particularly likely to want only limited information, although this group consisted of only two patients and four care partners.

### Experiences and preferences with regard to communicating uncertainty about the cause of dementia

1. *Different levels of experienced uncertainty over time*

Before, during and after the diagnostic trajectory, participants experienced varying levels of uncertainty. Many participants reported having felt uncertain while awaiting diagnosis. For some of them, this period of uncertainty even extended over several years. The uncertainty reportedly resulted in many negative feelings like anger, restlessness, worry, overthinking and denial, which sometimes affected their daily functioning.

*Interviewer: “How was this period of uncertainty for you?”*

*Care partner: “Track 2, I'll just call it. That was the worst, because then you get the doubt of Oh…* *Because, you still don't know what is going on […] Yes, I found that a very stressful period.” (dementia due to FTD, care partner)*

*“I have actually been in doubt for 2.5 years. [….] I was really filling in things myself. If I didn't feel well […] I have good days, bad days and you know, I was really struggling with that [. ..] And well, I was also very busy at work because…..* *I sometimes forgot things. […] I often said to my colleague: Am I forgetting something? Or if I'm not doing something right, or if I'm doing something weird, then you have to say it, you know? It was like that all the time” (MCI due to VaD, patient)*

A few participants emphasized that the uncertainty also fostered a sense of hope, as it allowed for the possibility that their symptoms were not indicative of dementia.

After receiving the test results and diagnosis, uncertainty generally decreased. Sharing and showing specific test results, as well as language use and non-verbal behavior of the clinician, contributed to participants’ feelings of certainty about the dementia cause.

*Interviewer: “And did those spinal fluid values provide any clarity?”*

*Care partner: “For us that is of course…* *more that it gives her [clinician] clarity which makes us feel a little more… Because of course she has to judge that […]. But that does give you the feeling that a diagnosis is not made just like that. Let's just say, she gave us that feeling that it was actually observable.” (dementia due to LBD)*

Following the diagnostic consultation, the majority of participants experienced feelings of uncertainty related to prognosis, regardless of their dementia cause. They expressed a need for more information about prognosis, but also acknowledged that clinicians may not be able to provide such information.

1. *Variance in need for information about uncertainty*

We found variation in participants’ information preferences about uncertainty related to the dementia cause. Whereas some participants reported wanting to be made aware of any uncertainty, others only wanted to receive information once it was certain. Care partners seemed more likely to want information about any uncertainty.

“*Well, I would just say it's all interesting…, I'd actually like to know as much as possible about what's going on. And even if doctors themselves disagree. Then I would like to know that too.” (Dementia due to AD, care partner)*

“*I can understand that they have been looking for a long time, so what is it? That they can't really put their finger on it, so I can imagine that the neurologist has this uncertainty. But I don't think you should burden the patient with that uncertainty.” (MCI due to VaD, patient)*

1. *Preferences regarding uncertainty communication*

When presenting participants the hypothetical scenarios of communicating uncertainty, several preferences were observed. First, many participants valued the three-step approach for communicating uncertainty (i.e., normalizing uncertainty, addressing patients’ emotions, and supporting with coping) because it included honest and empathic communication. However, when the degree of perceived uncertainty was high, which was frequently the case in the third scenario [uncertainty due to ambiguous test results], the three-step approach often led participants to report feelings of frustration and lack of support. In addition, one patient mentioned that empathic communication by the clinician should remain restricted since the relationship with the clinician is a formal one, and therefore it may be more reassuring if the doctor remains rather factual.

Second, participants unanimously felt that a detailed explanation of uncertainty in the form of percentages for each of the suspected causes had no added value. Instead, they indicated a need for more information about what each cause entailed in terms of perceived symptoms and associated test results. In contrast, the detailed explanation in scenario 3 [specifying the test results, including their ambiguity] was appreciated by a few participants as it gave them slightly more information about the clinician's considerations.

Third, some participants suggested clinicians could provide information about uncertainty in chunks, and check participants’ understanding, as well as provide the opportunity to ask questions during the consultation and in follow-up meetings. Finally, the importance of understandable communication and transparency about the uncertainty was often mentioned, illustrated by the following quote:

“*Yes, then they should just say that, we will never fully find out, but be honest, just like he was honest then or said that about the semantic dementia. Yeah, we don't 100% know everything about that, so yeah, well. Then it is what it is, […] but that's just possible sometimes. Yes, not everything is always 100% predictable.” (FTD, patient)*

Notably, this quote also includes the perception that certainty about the dementia cause could not always be obtained, which was supported by some participants:

“*But I don't think you'll know about Alzheimer's or FTD until you've died and put the brain under the microscope, then you will know for sure, right?” (FTD, care partner)*

Contrastingly, other participants assumed that specialized hospitals could eventually always determine the cause, for example by performing additional tests.

“*Yes, sorry, that is of course not an academic hospital. That might be in a [non-academic] hospital or something, but if that is the case, then they should indeed refer you to an academic hospital, where they can do those tests.” (mixed pathology (AD & possibly LBD, care partner)*

“*That's what I'm saying, we can even fly to the moon, I mean, it doesn't end here, I guess.” (MCI due to VaD, patient)*

## Discussion

In this qualitative study, we explored the views of patients with MCI or dementia and their care partners regarding communication about the cause of dementia and its associated uncertainties. We found that patients and care partners had limited understanding of the meaning of a dementia cause and that their preferences regarding receiving etiological information varied. Some indicated a high need for information because such information provided clarity and confirmation and enabled appropriate care. Others did not want to receive information about the cause as they perceived limited value in addition to the broad clinical syndrome diagnosis in terms of treatment and prognosis and feared information overload. In terms of the best strategies to communicate etiological information, participants valued clinicians’ straightforward communication, providing visualizations of test results, and explaining how test results related to their dementia cause. Information needs regarding clinicians’ communication of uncertainty also varied yet care partners especially preferred to receive uncertain information. Most participants appreciated a three-step approach for communicating uncertainty (normalizing uncertainty, addressing patients’ emotions, and supporting with coping), including honest and empathic communication. A detailed explanation of uncertainty was generally not valued.

Patients’ and care partners’ limited understanding about the dementia cause in our research resonates with findings of other studies.^18,48^ These showed patients and care partners to lack understanding of dementia being a syndrome with varying underlying etiologies, and perceiving AD as worse than dementia. We additionally found that patients and care partners often saw no benefit in determining the cause of dementia in view of treatment and prognosis, without realizing such information in practice could matter for receiving appropriate care.^5^ This finding underlines the importance of educating patients about the meaning and value of the dementia cause, to allow them to reach a well-informed decision about whether to undergo extensive diagnostic testing. When patients and care partners, after explaining the potential harms and benefits, still do not desire to receive information about the cause, it should be questioned whether performing certain diagnostic tests is warranted. When determining the cause nevertheless remains relevant from the clinician's perspective, a consideration could be, in agreement with the patient, to not disclose specific test results in much detail.

Many participants often spontaneously mentioned how reading information in the medical record or online prior to the diagnostic consultation could cause confusion and induce feelings of uncertainty. Literature in the field of oncology highlights similar potential negative consequences of searching for such information, although potential advantages were mentioned also, like facilitating preparation for consultations and reducing anxiety because of shorter time awaiting test results.^49–52^ Nevertheless, given the negative experiences of our participants, one recommendation for clinical practice would be to inform patients and care partners about the potential consequences of having insight in their test results when entering the diagnostic trajectory. Another recommendation is to inquire during the diagnostic consultation whether patients and care partners have seen their test results or searched for online information, and whether this resulted in any questions. An additional finding of our study was that particularly care partners were consulting the medical record and online information, which aligns with a study reporting that care partners searched for information on the internet, while people with dementia asked their family for information.^53^ Hence, different approaches to fulfill the information needs of both patients and care partners may be required. One could for example schedule an additional consultation, with solely the care partner, to discuss any remaining questions, and/or written information could be provided.

Our findings reveal considerable variation in preferences of patients and care partners regarding what information to provide about uncertainty related to the cause of dementia, as well as how to communicate this uncertainty. Many participants did not value a detailed explanation of uncertainty by providing percentages of the suspected contribution of each cause. Although the three-step approach for communicating uncertainty (i.e., normalizing uncertainty, addressing patients’ emotions, and supporting with coping) was valued by many, when the degree of uncertainty was high, this approach seemed to cause feelings of frustration and lack of support instead of providing clarity. Participants then rather wished to receive information about what next steps to take to obtain more certainty. All in all, these findings emphasize that there is not one best approach for communicating uncertainty related to the cause of dementia. The current literature also does not provide a solid answer. Therefore, further research is needed to investigate the effects of different communication approaches on understanding, recall and acceptance of uncertainty information, and to assess to what extent and how communication preferences differ between patients and care partners. As further developments in diagnostic testing and identification of the cause of dementia are expected, such insights will become increasingly important.^54^ Additionally, our findings illustrate that some patients and care partners incorrectly perceived that clinicians are always capable of determining the exact cause and obtaining complete diagnostic certainty, likely because of the extensive diagnostic test battery that they underwent. However, because of the complexity and ambiguity that are associated with novel diagnostic tests and frequently occurring mixed pathologies, uncertainty is often a reality of clinical practice.^15^ In addition, the progressive yet unpredictable nature of the disease(s) comes with inherent uncertainty, so regardless of knowing the cause, feelings of uncertainty will remain when confronted with MCI or dementia.^16,55^ These aspects highlight the importance of managing patients’ and care partners’ expectations at the start of the diagnostic trajectory, by explaining that complete diagnostic certainty cannot (always) be obtained, as well as exploring ways to help them cope with uncertainty.

A strength of our study is the recruitment strategy specifically focusing on patients with different dementia causes to explore whether their understanding and preferences differed. With the exception of (care partners of) patients with mixed pathology, who appeared to have somewhat higher information needs about the cause, we observed no differences between patients with different dementia causes. This may partly be due to the small number of participants included per dementia cause. Further larger-scale research is needed to confirm these findings. Another strength is the use of hypothetical, yet clinically valid scenarios of uncertainty, which allowed patients and care partners to reflect on their own experiences. These scenarios included simplified information about dementia causes by only focusing on AD, LBD, FTD, and VaD, but still revealed considerable variation in participants’ information needs. In practice, patient information needs may vary even more, especially when considering that certain dementia causes also consist of different clinical syndromes with distinct prognoses and care needs. Our study also had some limitations. The majority of our participants were recruited from a tertiary memory clinic specialized in young-onset dementia and with an extensive diagnostic work-up, which attracts a specific population.^30^ Half of the participants visited the hospital for a second opinion, which may have resulted in higher information needs regarding the cause. Moreover, information needs may have been impacted by the Dutch context and culture, as amyloid targeting therapies are not yet available and therefore determining the cause of dementia is of less importance. In addition, self-selection biases may have occurred, e.g., participants may have had a more positive experience than those who did not volunteer for the study. Furthermore, since only a few participants from non-academic hospitals were included, their views may be less represented in our study. Additionally, although we took much effort to facilitate focus group sessions, we could only perform one (with patients with MCI and dementia due to AD) since non-AD causes are diagnosed less frequently, making it impossible to include enough patients who had received their diagnosis recently. Finally, in multiple interviews the care partner predominantly was speaking, which may have resulted in the patient perspective being less represented.

### Conclusion

This article provides insight into the perspectives of patients with MCI or dementia and their care partners, on what communication they prefer regarding the cause of dementia and related uncertainty. High variation in information and communication needs regarding the dementia cause and uncertainty was found, highlighting the need for a tailored approach. Additionally, clinicians should support patients and care partners in the decision-making process and in understanding and coping with the information, by informing them beforehand about the potential relevance of obtaining the cause, as well as by managing their expectations about the likelihood of obtaining diagnostic certainty. Future research investigating which approaches for communicating diagnostic uncertainty work best for information retention and psychological wellbeing is recommended. Moreover, differences in communication preferences between patients and care partners, dementia causes, hospitals (including whether or not patients visited for a second opinion) and between various cultures, could be determined on a larger scale.

## Supplemental Material

sj-docx-1-alz-10.1177_13872877261435882 - Supplemental material for Patients’ and care partners’ views on communicating the cause of dementia and related uncertainties: A qualitative study Supplemental material, sj-docx-1-alz-10.1177_13872877261435882 for Patients’ and care partners’ views on communicating the cause of dementia and related uncertainties: A qualitative study by Jolanda H. M. Dobbe, Leonie N. C. Visser, Tim I. Waagemans, Geert J. Biessels, Flora H. Duits, Marij A. Hillen, A. W. Evelien Lemstra, Gerwin Roks, Inez H. G. B. Ramakers and Ellen M. A. Smets in Journal of Alzheimer's Disease

sj-docx-2-alz-10.1177_13872877261435882 - Supplemental material for Patients’ and care partners’ views on communicating the cause of dementia and related uncertainties: A qualitative study Supplemental material, sj-docx-2-alz-10.1177_13872877261435882 for Patients’ and care partners’ views on communicating the cause of dementia and related uncertainties: A qualitative study by Jolanda H. M. Dobbe, Leonie N. C. Visser, Tim I. Waagemans, Geert J. Biessels, Flora H. Duits, Marij A. Hillen, A. W. Evelien Lemstra, Gerwin Roks, Inez H. G. B. Ramakers and Ellen M. A. Smets in Journal of Alzheimer's Disease
