# Supplementary material for: Patients’ and care partners’ views on communicating the cause of dementia and related uncertainties: A qualitative study
Source: J Alzheimers Dis. 2026 Apr 3;111(2):746–56. doi: 10.1177/13872877261435882 (PMC13161501; doi:10.1177/13872877261435882)
Supplement: sj-docx-1-alz-10.1177_13872877261435882 - Supplemental material for Patients’ and care partners’ views on communicating the cause of dementia and related uncertainties: A qualitative study [file sj-docx-1-alz-10.1177_13872877261435882.docx]

**Supplemental File 1: Topic guide**

Part 1

What has been told to you about the cause of the complaints and how has that been told?

- What do you think of when we talk about 'the cause of the complaints'? What do you mean by that?
- What exactly did the doctor tell you about the cause of the complaints?
- What words did the doctor use to explain that?
- In addition to the information that the doctor told you verbally, did you also receive an explanation on paper, or did the doctor show you something (brain scan, for example)?

Statements

1. The doctor gave me more information about the cause of my complaints than I could understand at the time.
   1. If agree: specify 'too much'. So what exactly was too much?
      1. Lots of results do discuss
      2. A lot of complicated information (so difficult to understand)
      3. It evoked many emotions
   2. If disagree: was it just enough, or too little?
   3. If disagree: did you miss information, and if so, what?

1. I found it easy to understand what the doctor said about the cause of my complaints. I was then able to explain it to others.
   1. If agree: How would you explain to others what the cause of your complaints is?
   2. If agree: Did the doctor do anything to make it understandable?
   3. If disagree: Can you tell us what you did not understand?
      1. Certain words the doctor used?
   4. If disagree:
      1. What makes you think you didn't understand?
      2. ii. Does it also have to do with the fact that the doctor has given you too much information?

1. The doctor was very certain about what caused my complaints.
   1. If agree: How did the doctor show that he or she was certain about the cause of the complaints?
      1. Used certain words?
      2. Body position
      3. Facial expression
      4. Hand gestures
   2. If disagree: How did the doctor describe this uncertainty?
   3. Both agree and disagree: What feeling did that [certainty/uncertainty] give you?

1. During the conversation, the doctor gave me the space to express my emotions about my complaints.
   1. If agree: How did the doctor give you space?
      1. Used certain words/said things
      2. Dropping silences (processing time)
      3. Recognizing emotions
   2. If agree/disagree: What did you think about that?
   3. If disagree: What could the doctor have done differently?

1. The doctor took the time to explain in detail the cause of my complaints.
   1. If agree: How did you notice that?
   2. If agree: What do you mean by detailed explanation?
   3. If agree: Can you give an example of detailed explanations given by the doctor?
      1. MRI-scan: explanation of the degree of brain shrinkage
      2. Blood tests: explanation what values and proteins mean and do
   4. If disagree: Can you explain what made you feel that way?
   5. If disagree: Can you give an example of how the doctor gave a very general explanation about the cause of the complaints?
      1. No precise explanation of what a certain value/observation means?
   6. If disagree: How would you have liked it to be different?

Part 2

Scenario 1:

1. What is your first reaction when you hear and read this?
2. What questions do you still have regarding this information?
3. What is the most important information you get from this?

Scenario 2:

1. What is your first reaction when you hear and read this?
   1. Would you like to receive this information [uncertainty]? Would you like to know?
2. What questions do you still have regarding this information?
3. What is the most important information you get from this?

When we add information:

- What do you think about this?
- What makes it complicated or not complicated?
  1. Do you understand what is being said?
- Preference for blue or orange? Or neither/a combination of both?
- Blue: what do you think of what the doctor is doing here?
  1. Do you like that the doctor acknowledges that the uncertainty is annoying?
  2. Is this kind of message useful to you?
  3. What do you think of the last sentence [best possible care]?
  4. What do you think of by the word ‘care'?
  5. If applicable, what would be better wording here?
- Orange: does this make it clearer? Why/why not?
  1. Precise numbers nice?
  2. How much exactly do you want to know?
  3. Suggestion for how the doctor can say it better?
- Is this too little/enough/too much information for you to understand what is going on?
- Is there information you are missing?

Scenario 3:

1. What is your first reaction when you hear and read this?
   1. Would you like to receive this information [uncertainty]? Would you like to know?
2. What questions do you still have regarding this information?
3. What is the most important information you get from this?

When we add information:

- What do you think about this?
- What makes it complicated or not complicated?
  1. Do you understand what is being said?
- Preference for blue or orange? Or neither/a combination of both?
- Blue: what do you think of what the doctor is doing here?
  1. What do you think about sharing the limitations of current testing?
     - And the way the doctor tells you this, is it clear?
  2. Do you feel that, as it is currently written, there are still opportunities for additional research? Or can there be no more certainty than there is now?
- Orange: does this make it clearer? Why/why not?
  1. What do you think of the word 'brain shrinkage'?
     - Is this something the doctor also used in your case, and if so, do you understand?
- Is this too little/enough/too much information for you to understand what is going on?
- Is there information you are missing?
