# Supplementary material for: Patients’ and care partners’ views on communicating the cause of dementia and related uncertainties: A qualitative study
Source: J Alzheimers Dis. 2026 Apr 3;111(2):746–56. doi: 10.1177/13872877261435882 (PMC13161501; doi:10.1177/13872877261435882)
Supplement: sj-docx-2-alz-10.1177_13872877261435882 - Supplemental material for Patients’ and care partners’ views on communicating the cause of dementia and related uncertainties: A qualitative study [file sj-docx-2-alz-10.1177_13872877261435882.docx]

**Supplemental File 2: Scenarios of communicating uncertainty**


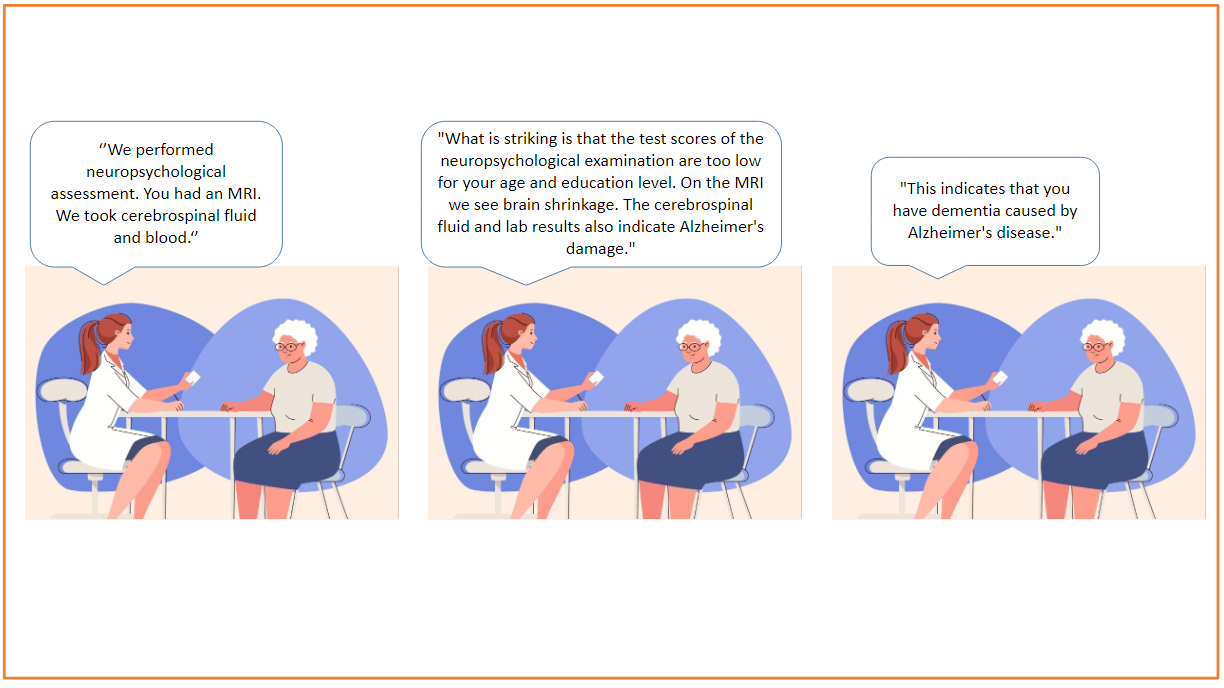


Figure 1. Scenario 1 (no uncertainty), Alzheimer’s disease.


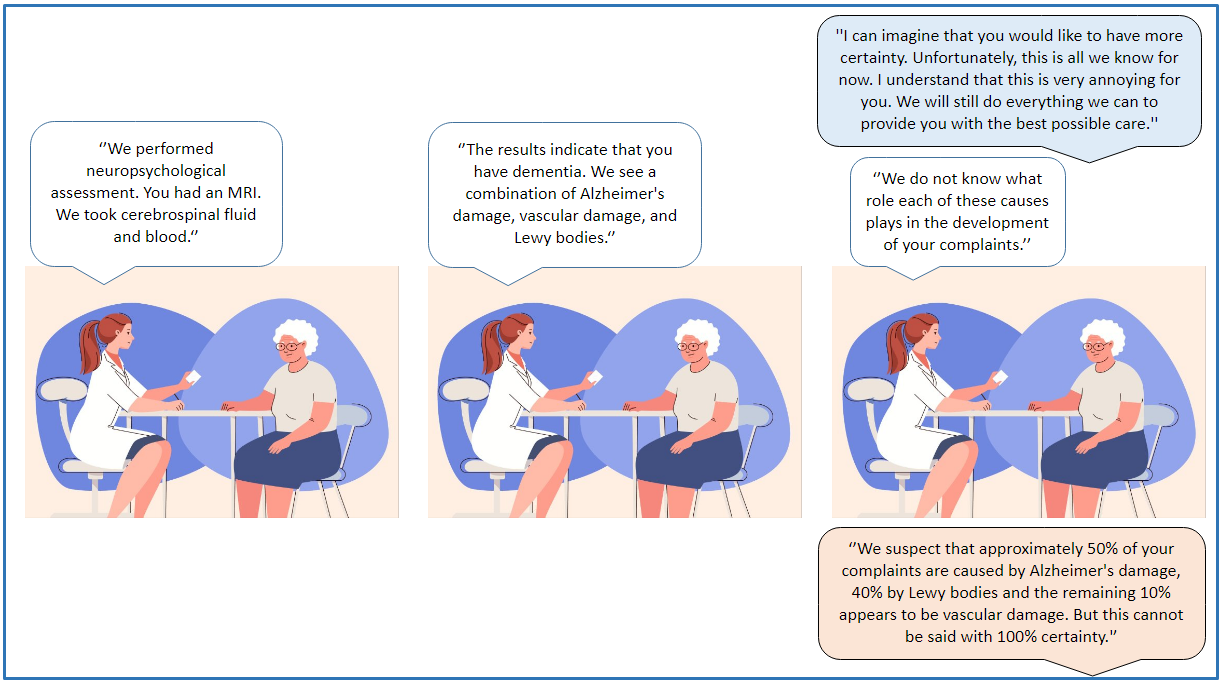


Figure 2. Scenario 2 (uncertainty due to complexity), Alzheimer’s disease. Blue speech bubble = three-step approach for communicating uncertainty; orange speech bubble = detailed explanation of uncertainty.


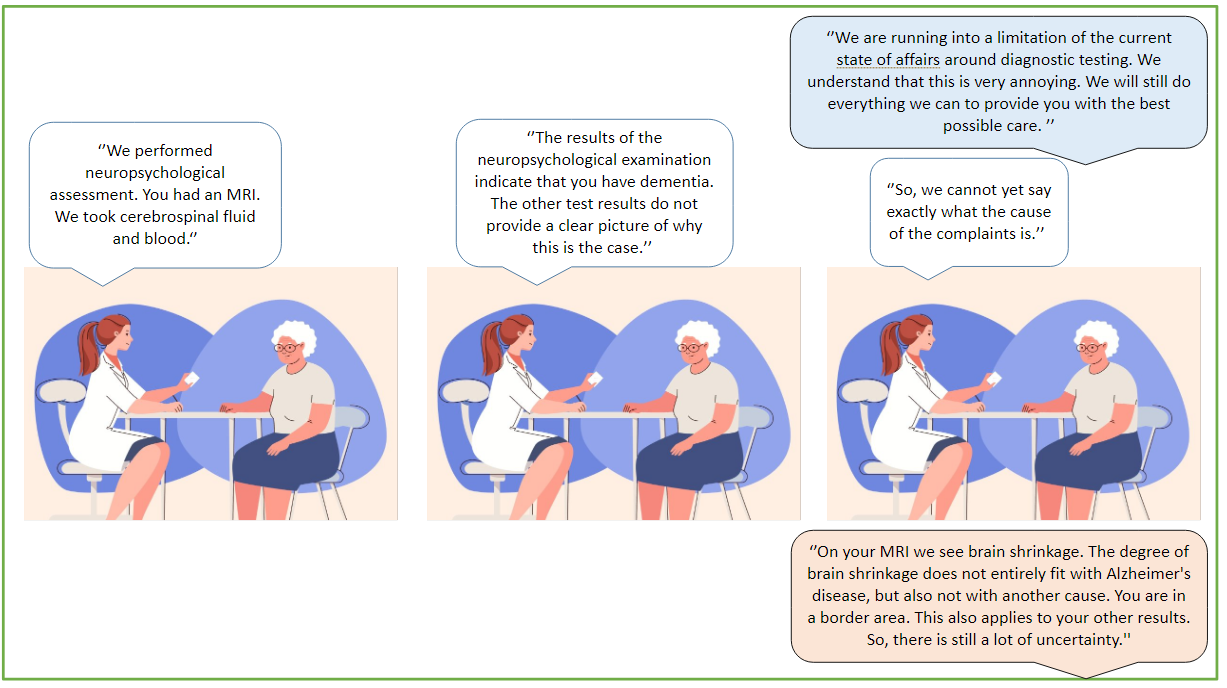


Figure 3. Scenario 3 (uncertainty due to ambiguity), Alzheimer’s disease. Blue speech bubble = three-step approach for communicating uncertainty; orange speech bubble = detailed explanation of uncertainty.
